# Adoption of a Data‐Driven Bayesian Belief Network Investigating Organizational Factors that Influence Patient Safety

**DOI:** 10.1111/risa.13610

**Published:** 2020-10-18

**Authors:** Mecit Can Emre Simsekler, Abroon Qazi

**Affiliations:** ^1^ Department of Industrial and Systems Engineering Khalifa University of Science and Technology Abu Dhabi UAE; ^2^ School of Management University College London London E14 5AA UK; ^3^ School of Business Administration American University of Sharjah Sharjah UAE

**Keywords:** Bayesian Network, healthcare operations, medical errors, patient safety, risk

## Abstract

Medical errors pose high risks to patients. Several organizational factors may impact the high rate of medical errors in complex and dynamic healthcare systems. However, limited research is available regarding probabilistic interdependencies between the organizational factors and patient safety errors. To explore this, we adopt a data‐driven Bayesian Belief Network (BBN) model to represent a class of probabilistic models, using the hospital‐level aggregate survey data from U.K. hospitals. Leveraging the use of probabilistic dependence models and visual features in the BBN model, the results shed new light on relationships existing among eight organizational factors and patient safety errors. With the high prediction capability, the data‐driven approach results suggest that “health and well‐being” and “bullying and harassment in the work environment” are the two leading factors influencing the number of reported errors and near misses affecting patient safety. This study provides significant insights to understand organizational factors’ role and their relative importance in supporting decision‐making and safety improvements.

## 1 INTRODUCTION

Medical errors pose significant risks to patients in healthcare. Since the U.S. Institute of Medicine (IoM) published their pioneer safety report, which shows up to 98,000 people dying as a consequence of preventable medical errors (Kohn, Corrigan, & Donaldson, 2000), substantial efforts have been made to improve the situation (Gandhi et al., 2018; Jones, Vaux, & Olsson‐Brown, 2019). However, recent studies still show that medical errors are among the leading causes of deaths, which only in the U.S. estimated over 250,000 deaths in a year (Makary & Daniel, 2016).

Such medical errors occur within the dynamic and complex healthcare delivery systems due to several organizational factors (Simsekler, Gurses, Smith, & Ozonoff, 2019). To identify such factors, earlier studies evaluated the working conditions as well as staff experiences and observations concerning teamwork (Wooldridge et al., 2020), communication (Peadon, Hurley, & Hutchinson, 2020; Wooldridge, Carayon, Shaffer, & Eagan, 2018), safety climate (Aljabri et al., 2020; Simsekler, 2019), risk practice (Kaya, Ward, Pearman, & Clarkson, 2019a, 2020), and near miss event reporting (Patterson & Pace, 2016), among others. While these studies focused solely on individual factors, they also highlighted possible dependencies among the factors within the complex systems. Earlier studies also noted that various measurement instruments are available (Kristensen, 2016) to explore the organizational factors, mainly in the form of cross‐sectional surveys (The Health Foundation, 2011). However, it is important to note that each tool has unique cultural domains, and they experience certain limitations when it comes to data validity and reliability (Campione, Yount, & Sorra, 2018; Meddings et al., 2017). Further, earlier studies revealed that the limited use of statistical approaches might have failed to identify complex relationships between multidimensional organizational factors and patient safety (Reis, Paiva, & Sousa, 2018).

These concerns have led us to adopt a data‐driven Bayesian Belief Network (BBN) model to help explore organizational factors that may influence patient safety. Being at the intersection between statistics and machine learning, a BBN model represents either causal probabilistic relationships or statistical dependencies among interconnected variables. Further, when BBN features are exploited, the analysis goes beyond basic data analysis and potentially reveals deeper insights, probabilistic outcomes, and valuable findings.

Identifying organizational factors that contribute to patient safety errors is vital toward knowing how to provide sustainable safety improvements. The primary purpose of this article is to evaluate the relationship between organizational factors and reported error rates related to patient safety and establish the relative importance of the factors through the lens of BBNs. In this study, our intention is not to test specific hypotheses, but rather to explore organizational factors and their probabilistic dependencies on the safety outcome.

The outline of the paper is as follows: The literature review on organizational factors and patient safety, and BBNs is presented in Section 2. The methodology is explained in Section 3. The results and interpretations are presented in Section 4. The implications of our study are discussed in Section 5. Finally, conclusions, study limitations, and future research directions are revealed in Section 6.

## 2 LITERATURE REVIEW

### 2.1 Organizational Factors and Medical Errors

Various factors may play a role in the occurrence of incidents in different industries and domains (Årstad & Aven, 2017; Aven & Ylönen, 2018). Healthcare is not exceptional where medical errors occur within complex healthcare delivery systems due to limited communication, safety culture, risk practice, among other factors (Ellahham, Ellahham, & Simsekler, 2019; Kaya, Ward, & Clarkson, 2019b; Meddings et al., 2017; Simsekler, Qazi, Alalami, Ellahham, & Ozonoff, 2020). Further, it should be noted that such factors may affect patients as well as healthcare providers. In fact, healthcare providers are at a higher risk of experiencing injuries than in many other industries (Aljabri et al., 2020). The providers are threatened by burnout, pain, and even the risk of disease or injury—factors that inhibit their ability to maintain consistent, high‐quality care (Dressner, 2017). While providers’ well‐being may trigger undesired safety‐related events, the fragile nature of safety culture may also be among the key factors that can influence safety errors (Flin, 2007).

Using surveys and questionnaires, valuable insights have been captured between the organizational factors and patient safety‐related measures (Patterson & Pace, 2016). For instance, Kristensen (2016) evaluated 33 questionnaires used for the safety culture measurement, including a range of organizational factors. Hospital Survey on Patient Safety Culture (HSOPSC) (Jones, Skinner, Xu, Sun, & Mueller, 2008), Patient Safety Culture in Healthcare Organizations (PSCHO) (Singer et al., 2007), Safety Attitudes Questionnaire (SAQ) (Sexton et al., 2006), Manchester Patient Safety Assessment Framework (MAPSAF) (Parker, Kirk, Claridge, Lawrie, & Ashcroft, 2007), Safety Organizing Scale (SOS) (Vogus & Sutcliffe, 2007), and British National Health Service (NHS) Staff Surveys (NHS SCC, 2019) are among the most common tools capturing the experience of healthcare providers to reveal insights on organizational factors in this particular healthcare safety context.

Among the most common approaches discussed above is HSOPSC used in over 60 countries (Blegen, Gearhart, O'Brien, Sehgal, & Alldredge, 2009) to measure teamwork, communication, manager support, and others (Waterson, Carman, Manser, & Hammer, 2019) as possible organizational factors affecting patient safety. A recent comprehensive review brought together various healthcare studies from 21 countries from 2007 to 2016, utilizing HSOPSC (Reis et al., 2018). The study showed significant variations in the results. While some studies concluded that “*teamwork within hospital units*” was the most important dimension (Blegen et al., 2009; Bodur & Filiz, 2010; Hellings, Schrooten, Klazinga, & Vleugels, 2007), others found “*organizational learning and continuous improvement*” to be more strongly correlated with safety errors (Al‐Ahmadi, 2009; Bagnasco et al., 2011; Nie et al., 2013). In contrast, according to the study by Abdelhai, Abdelaziz, and Ghanem (2012), “*overall perceptions of patient* *safety*” was the most prominent dimension.

The studies discussed above reveal that various organizational factors play a role in the patient safety context underlying medical errors. Therefore, healthcare provider experience as a component can comprehensively capture such factors within the complex healthcare environments. Recent studies support this idea by suggesting that provider experience needs to be addressed due to its close association with the quality of care and safety (Lee, Seo, Hladkyj, Lovell, & Schwartzmann, 2013). It is also worth noting that given the complex and dynamic nature of healthcare systems, the use of experience can allow for the identification of safety beliefs, attitudes, and workforce behaviors thoroughly (Kouabenan, Ngueutsa, & Mbaye, 2015; Liu et al., 2015). Moreover, staff experience may also identify the root causes that trigger safety outcomes, such as incidents and injuries (Fernández‐Muñiz, Montes‐Peón, & Vázquez‐Ordás, 2007).

The systematic review by Reis et al. (2018) also reveals the limited nature of the statistical analyses of healthcare safety data. For instance, only two of the 33 surveys (6%) investigated the effect of multiple survey dimensions on the patient safety using regression models (Al‐Ahmadi, 2009; Smits et al., 2012). While Al‐Ahmadi (2009) used a linear regression analysis to investigate the association between the event reporting and safety culture components, Smits et al. (2012) analyzed the data using a multilevel logistic regression analysis. It must be stressed that most studies focused solely on a single organizational factor or some without simultaneously considering all factors in a probabilistic setting. Therefore, their potential interdependencies have not been discussed with the use of probabilistic and graphical models. In order to explore this, the use of powerful tools, such as BBN models, may significantly help with predicting the relationships as well as identifying the relative importance of organizational factors to the patient safety outcomes.

### 2.2 Bayesian Belief Networks

BBNs are probabilistic graphical models that can quantify and display complex relationships among variables (Salini & Kenett, 2009; Sener, Barut, Dag, & Yildirim, 2019a). A BBN model comprises a set of nodes representing variables and arcs that either represent causal relationships or statistical dependencies among interconnected variables (Hanea, McBride, Burgman, & Wintle, 2018). The strength of dependenices among interconnected variables is captured in the form of probability distributions. In earlier studies, BBN models have been used in a wide range of application areas such as project management (de Oliveira, Possamai, Dalla Valentina, & Flesch, 2012), business ethics (Ekici & Önsel, 2013), supply chain management (Sener, Barut, Oztekin, Avcilar, & Yildirim, 2019b), new product development (Chin, Tang, Yang, Wong, & Wang, 2009), and others. BBNs have been extensively explored in decision‐making under risk and uncertainty due to their ability to generate and assess the impact of different what‐if scenarios. Some of the risk and uncertainty‐related application areas include eruption forecasting (Christophersen et al., 2018), climate change assessment (Hanea et al., 2018), coastal risk analysis (Jäger, Christie, Hanea, den Heijer, & Spencer, 2018), risk assessment in process plants (Ale et al., 2014), and others (Adedipe, Shafiee, & Zio, 2020; Aven, 2016).

Earlier studies identified a range of benefits associated with the use of BBNs (Cugnata, Kenett, & Salini, 2016). First, the graphical representation of the problem provides an opportunity for stakeholders to visualize complex interactions among different variables (Hanea, Kurowicka, & Cooke, 2006). Second, probabilistic reasoning, a key notion in risk and uncertainty analysis (Aven, 2017, 2018; Shortridge, Aven, & Guikema, 2017), is captured in a simplistic manner and distributed through the model, and past beliefs on uncertain variables are efficiently updated with the evidence against distinct sources in the network (Jäger et al., 2018). Third, uncertainty in reasoning is considered, and the (in)dependence relationships are identified (Qazi, Dikmen, & Birgonul, 2020a). All these can be achieved with even limited empirical data (Hanea et al., 2006). Despite these benefits, there are some shortcomings of the method to note. For instance, when data are not available, the development and quantification of a model using expert judgment is quite challenging (Hanea, Morales Napoles, & Ababei, 2015; Werner, Bedford, Cooke, Hanea, & Morales‐Nápoles, 2017; Hanea et al., 2018). Another limitation relates to avoiding any feedback loops in the model, implying that the BBN model must be structured as an acyclic graph (Nielsen & Jensen, 2007). Moreover, the software used to develop the network may have a limited capability to work with continuous variables, which might then be discretized; therefore, this may capture limited information on the original variable distribution (Qazi et al., 2020a).

BBNs provide a visual ability for users and decision‐makers to establish the propagation impact of variables in a network setting (Lawrence, Ibne Hossain, Jaradat, & Hamilton, 2020). For instance, BBN models can help visualize how a variable influences other variables across a network in the event of its mitigation or realization (Kelangath, Das, Quigley, & Hirdaris, 2012). This assessment of the network‐wide propagation impact of individual variables can lead to prioritizing and managing critical variables (Kabir & Papadopoulos, 2019). Both expert judgment and data can be utilized for developing a BBN model (Hanea et al., 2018). In this article, we use a data‐driven approach for developing a BBN model.

While BBNs have been successfully explored in various application areas, their potential contribution has not been evaluated in the patient safety context yet. It is, therefore, aimed to explore the application of BBNs in the patient safety field with a unique survey data‐set. Using the research methodology in Section 3, our goal is to identify the probabilistic dependencies among organizational factors and patient safety errors.

## 3 METHODOLOGY

### 3.1 Data Sources

Our data source is the British NHS staff survey data. With the availability of the data, we analyzed the following eight factors in the survey that could be related to patient safety: (X1) *equality, diversity, and inclusion;* (X2) *health and well‐being;* (X3) *immediate managers;* (X4) *quality of care;* (X5) *safety culture;* (X6) *team working;* (X7) *safe environment—bullying and harassment (b&h)*; and (X8) *safe environment—violence*. Within these factors, there are 32 specific survey items. Table I lists all the factors, along with the patient safety error variable. Further information on the specific survey items can be found in Appendix Table AI.

**Table I:** List of Organizational Factors Influencing Patient Safety Errors

ID	Organizational Factors	Unit/Remarks
X1	Equality, diversity, and inclusion	0–10 pt scale
X2	Safety culture	0–10 pt scale
X3	Health and well‐being	0–10 pt scale
X4	Immediate managers	0–10 pt scale
X5	Quality of care	0–10 pt scale
X6	Team working	0–10 pt scale
X7	Safe environment—bullying and harassment (b&h)	0–10 pt scale
X8	Safe environment—violence	0–10 pt scale
X9	Target variable ‐ Patient safety errors	% Yes

As shown in the unit/remarks column in Table I, all factors (X1–X8) are scored on a scale between 0 and 10 points, which is primarily made available by NHS for hospitals to benchmark their grades with others (NHS SCC, 2019). Responses for the contributing questions from each hospital were rescored to fit this scale by NHS. Each theme within the survey representing an organizational factor (see Table I) has a unique calculation. For instance, *equality, diversity, and inclusion* (X1) has been fed by four questions (see Table A.I). All participants who have responded to two of the questions or more get allocated an overall score that is the average score of their rescored questions. Each organizational factor's score is the average score of all participants’ overall scores (NHS SSCC, 2019). As the outcome measure, we use the same survey data on the reported number of safety incidents that could have harmed patients (X9, item *q16b*). The calculation of this outcome measure is based on the percentage of staff selecting “yes” out of those who responded to the question from the respective hospital. It should be noted that a higher score provides a more favorable result for organizational factors, while it is the opposite for the target variable (patient safety errors).

It should be noted that hospitals may have different characteristics; therefore, hospital‐level comparison may be limited in prediction and feature importance analyses. However, to prevent such issues, the survey data have primarily been weighted by NHS considering the occupational group weight, trust size weight, and the combination of these two weights (NHS SCC, 2019). The weighting procedure aims to ensure that no organization appears worse or better than others due to disparities in trust sizes and occupational groups. For instance, the calculation for trust size weight is made for all trusts participating in the study. The trust size weight is calculated by dividing the total number of eligible staff by the number of respondents. For the calculation of occupational group weight, the first step is to calculate the proportion of each occupational group within each trust. The second step is to calculate the average proportion of each occupational group within each benchmark group. The third and last step is to divide the benchmark group proportion (step 2) by the trust proportion (step 3). Finally, the combined weight is calculated by multiplying the occupational group weight with the trust size weight. Further information and examples on the staff survey methodology and methods used for weighting scores can be found on the NHS Staff Survey website (NHS SCC, 2019).

Table II shows the descriptive statistics of the organizational factors and target variable considered in this study.

**Table II:** Descriptive Summary for Organizational Factors and Target Variable

ID	Variable	Count	Mean	Median	*SD*	Min	Max	Range
X1	Equality, diversity, and inclusion	413	9.05	9.11	0.26	8.07	9.56	1.49
X2	Safety culture	413	6.60	6.61	0.25	5.68	7.23	1.55
X3	Health and well‐being	413	5.95	5.95	0.29	5.19	6.78	1.59
X4	Immediate managers	413	6.71	6.71	0.23	6.00	7.41	1.41
X5	Quality of care	413	7.46	7.45	0.21	6.67	8.20	1.53
X6	Team working	413	6.53	6.52	0.20	5.87	7.17	1.30
X7	Safe environment—b&h	413	7.91	7.95	0.27	6.99	8.49	1.50
X8	Safe environment—violence	413	9.40	9.34	0.09	9.13	9.67	0.54
X9	Patient safety errors	413	0.29	0.29	0.03	0.19	0.39	0.20

As shown in Table III, the intercorrelation analysis of organizational factors using the Spearman's coefficient was also conducted to explore the statistical properties and evaluate the strength of association between the factors.

**Table III:** Intercorrelation of Organizational Factors Using Spearman's Correlation

ID	Variable	X1	X2	X3	X4	X5	X6	X7	X8
X1	Equality, diversity, and inclusion	1							
X2	Safety culture	0.14	1						
X3	Health and well‐being	0.51	0.54	1					
X4	Immediate managers	0.27	0.78	0.60	1				
X5	Quality of care	‐0.10	0.57	0.42	0.42	1			
X6	Team working	0.25	0.73	0.61	0.82	0.52	1		
X7	Safe environment—b&h	0.67	0.47	0.70	0.53	0.17	0.47	1	
X8	Safe environment—violence	0.01	0.20	0.04	0.10	−0.01	0.10	0.11	1

As shown in Table IV, a correlation analysis was carried out to examine the statistical properties and test each coefficient's significance using *p*‐values. Results showed that the relationships between each organizational factor and patient safety errors are statistically significant (see Table IV). Results also showed that there is a negative correlation between the individual factors and the target variable.

**Table IV:** Correlation Strengths Between Organizational Factors and Patient Safety Errors

No	Organizational Factors	Spearman's Coefficient
X1	Equality, diversity, and inclusion	−0.36***
X2	Health and well‐being	−0.58***
X3	Immediate managers	−0.17***
X4	Quality of care	−0.35***
X5	Safety culture	−0.22***
X6	Team working	−0.21***
X7	Safe environment—b&h	−0.49***
X8	Safe environment—violence	−0.10***

*** *p* < 0.01,

** *p* < 0.05,

* *p* < 0.1.

### 3.2 Procedure

As discussed earlier, we adopt a data‐driven BBN model to investigate the probabilistic dependencies between the organizational factors and patient safety errors. A BBN model comprises the following elements:

- a set of variables along with directed edges between variables forming a directed acyclic graph (Shakeri, Zarei, Azar, & Maleki Minbash Razgah, 2020). A directed graph is acyclic if no directed path is available: $X_{1}\to \text{…}\to X_{n}$ so that $X_{1}=X_{n}$. Further, the directed edges represent statistical associations in a data‐driven BBN as the primary approach in developing the model in this study.
- a conditional probability table $P(X_{i}|pa\left(X_{i}\right))$ attached to each variable $X_{i}$ with parents $pa(X_{i})$. For instance, if there is an arc leading from each of $X_{2}$, $X_{7},$ and $X_{8}$ (organizational factors) to $X_{9}$ (target variable), $X_{2}$, $X_{7}$, and $X_{8}$ are classified as the parents of $X_{9}$.

In the BBN with a specification over $U\;=X_{1}\;,\text{…},X_{9}$, factoring joint probabilities are obtained through the chain rule of probability theory resulting from the calculations conducted under certain probability states. The value of a particular node is conditional solely on its parent nodes’ values in the BBN model. Thus, the unique joint probability distribution, P(U) represents the product of all conditional probability tables with the given Equation (1) below:

(1) $$P\left(U\right)=\prod_{i=1}^{9}P\left(X_{i}|\mathrm{pa}\left(X_{i}\right)\right)\;$$

The adoption of a discrete data‐driven BBN model includes the discretization of the survey data, selection of states for each organizational factor and patient safety errors, and establishing the strength of interdependency across interconnected variables. Many software packages, such as Hugin, AgenaRisk, Netica, and GeNIe, are available to adopt a data‐driven approach (Cox, Popken, & Sun, 2018) while using several algorithms such as Naive Bayes, Bayesian Search (BS), PC, and Greedy Thick Thinning (GTT), among others (BayesFusion, 2017; Kelangath et al., 2012). These algorithms can be classified into constraint‐based and score‐based methods. The constraint‐based methods, such as the PC algorithm, employ conditional independence statements to generate a network using data (Kelangath et al., 2012). On the other hand, the score‐based methods, such as the GTT algorithm, create and score several network structures and finally select the structure with the highest score (Ekici & Önsel Ekici, 2019). The prediction accuracy of such data‐driven models (Ekici & Önsel Ekici, 2019; Kelangath et al., 2012) can be ascertained using the Equation (2) below:

(2) $$Accuracy\;=\frac{number\;of\;correct\;predictions}{total\;number\;of\;records}\;$$

We developed our model in GeNIe 2.0 while adopting a uniform width discretization scheme, as proposed by Ekici and Önsel Ekici (2019). Further, we tested three different algorithms utilizing a mix of constraint‐based algorithm (PC) and score‐based algorithms (GTT and BS), in two various discretization schemes, two and three states. This procedure was adopted to establish the sensitivity of the results to the choice of algorithms and discretization states and to optimize the model for further analysis. As a result of this, the confusion matrix was constructed for the model providing the best possible prediction capability. A confusion matrix is an output of a validation process that represents the relationship between actual and predicted states associated with a model. The BBN model was validated through a *k*‐fold cross‐validation scheme (Marcot & Hanea, 2020) available in GeNIe (BayesFusion, 2017) that includes dividing a data‐set into *k* parts of equal size, training the network developed on *k*‐1 parts, and testing it on the last *k*th part. The process is repeated *k* times with a different part of the data selected for each testing iteration (Qazi, Dikmen, & Birgonul, 2020b). The results were found to be insensitive to the increase in *k* beyond 6. Following this, the discrete probability distribution of factors influencing patient safety errors was represented in the BBN model.

The results of the model and relative importance of factors contributing to the patient safety errors are presented in further detail in the next section.

## 4 RESULTS

**Fig 1:**
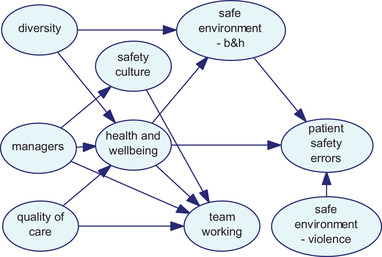
Network structure developed using the PC algorithm.

**Fig 2:**
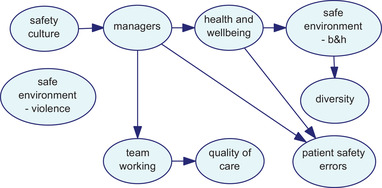
Network structure developed using the BS algorithm.

**Fig 3:**
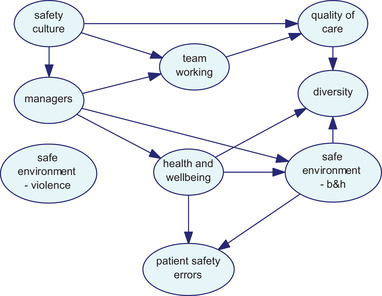
Network structure developed using the GTT algorithm.

As discussed in the procedure above, different network structures were developed using three algorithms. Figs. 1, 2, and 3 show the network structures related to the use of PC, BS, and GTT algorithms, respectively. As shown in each figure, the algorithms presented nine variables, including organizational factors and patient safety errors, with various arcs.

To test the effectiveness of the algorithms on the chosen data‐set, their prediction accuracy was calculated for the chosen two‐ and three‐state discretization schemes such that each variable was discretized into two and three states, respectively. As shown in Table V, PC outperformed the other two algorithms with the 78% prediction accuracy, one of the earliest and most popular algorithms introduced by Spirtes, Glymour, and Scheines (1993). Therefore, the remaining procedure is adopted through this specific algorithm in a three‐state discretization scheme. Although the prediction accuracy of PC was not significantly greater compared to other algorithms, it was deemed appropriate to utilize the same algorithm‐based model in our analysis due to its successful adoption in other application areas (Kelangath et al., 2012; Qazi & Dikmen, 2019).

**Table V:** Sensitivity of the Prediction Ability of Models Relative to Different Discretization Schemes and Algorithms

Discretization Scheme (Number of States)	Algorithm	Prediction Accuracy (In Percentage)
Two states	PC	74.1
	GTT	73.8
	BS	74.3
Three states	**PC**	**78.0**
	GTT	74.1
	BS	74.1

The confusion matrix in Table VI shows the association between the actual and predicted states for the PC algorithm in detail. The numbers shown in bold represent the correct predictions. For instance, out of 413 records tested in the validation stage, 322 were correctly diagnosed with an accuracy of 78%.

**Table VI:** Confusion matrix for the PC algorithm‐based network structure with three states for each variable

	Predicted		
	s1	s2	s3

Actual			
s1	**3**	48	0
s2	2	**303**	2
s3	0	39	**16**

The probability distribution of organizational factors associated with patient safety errors is shown in Fig. 4b. Regarding this model, it can be said that 13% of the cases were associated with the state 3 (high) of patient safety errors. The underlying distribution was highly skewed in the case of *diversity* and *safe environment—b&h*, while others were normally distributed. The model (see Fig. 4b) was then analyzed for the low (see Fig. 4a) and high state (see Fig. 4c) of patient safety errors to evaluate the change across variables in the network.

Fig 4(a) Backward propagation of beliefs once the low state of patient safety errors is established. (b) Probability distribution of factors associated with patent safety errors. (c) Backward propagation of beliefs once the high state of patient safety errors is established.

**Figure:**
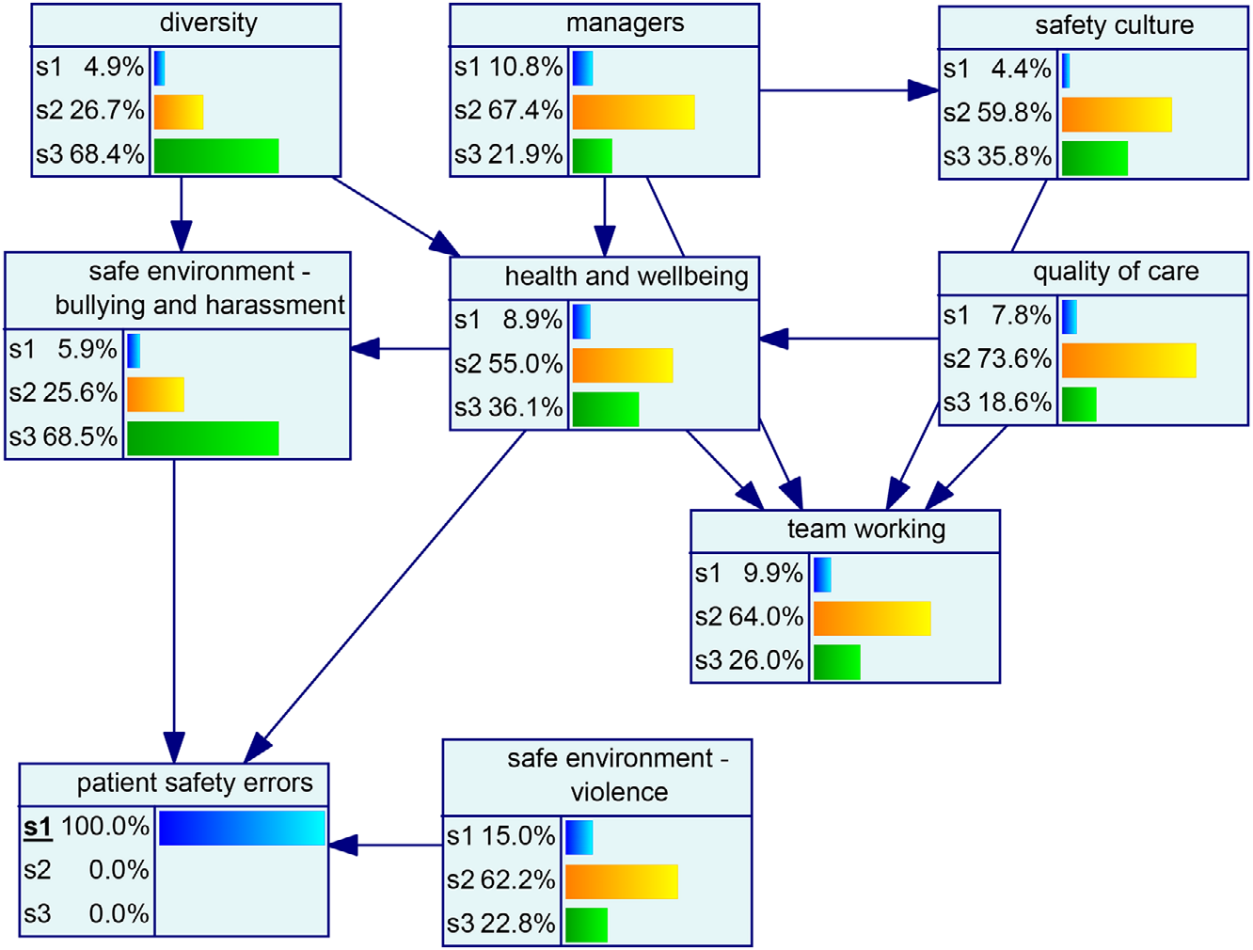

**Figure:**
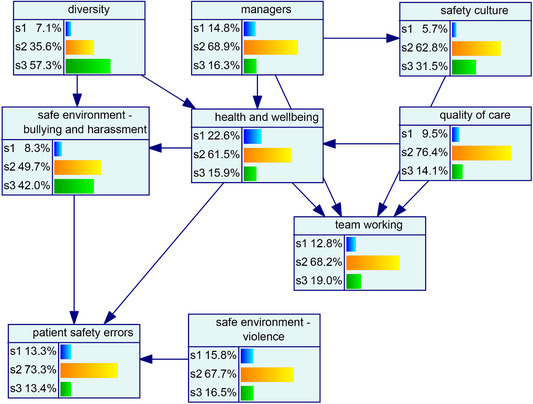

**Figure:**
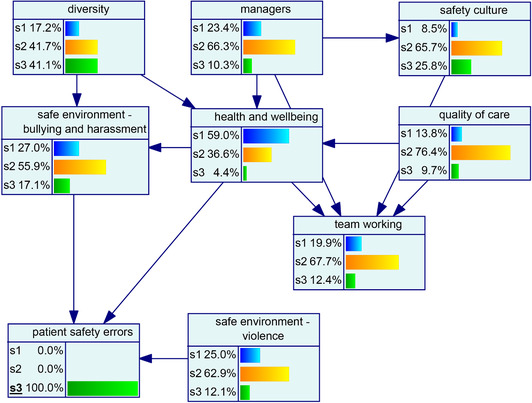

**Fig 5:**
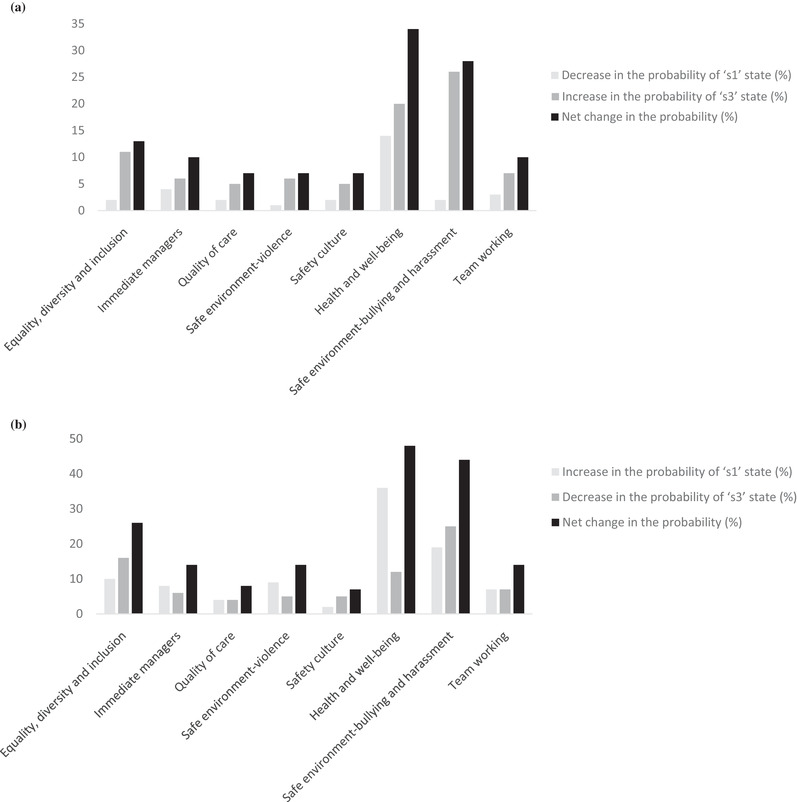
(a) Back propagation impact assessment given the patient safety errors in “s1” state. (b) Back propagation impact assessment given the patient safety errors in “s3” state.

The impact assessment across the organizational factors given the low and high state of patient safety errors is summarized in Fig. 5a and Fig. 5b, respectively. Overall, results showed that “*health and well‐being*” and “*safe environment—bullying and harassment*” are the two leading critical factors in the back propagation impact assessment given the low and high state of patient safety errors.

**Fig 6:**
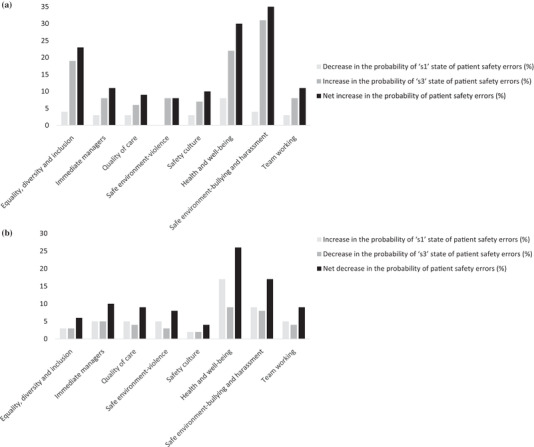
(a) Impact of individual factors on patient safety errors relative to their “s1” state. (b) Impact of individual factors on patient safety errors relative to their “s3” state.

The relative importance of individual factors was also established by assessing the change in the probability distribution of patient safety errors concerning the realization of each factor's extreme states. Vulnerability (resilience) of a factor represents its impact on a target variable associated with the realization of the worst (best) possible state of the factor (Hosseini & Ivanov, 2019). The assessment of the vulnerability and resilience potential of individual factors can help prioritize factors for developing strategies to enhance (mitigate) resilience (vulnerability) (Aven, 2011, 2019). “*S* *afe environment—bullying and harassment*” was identified as the most critical factor relative to its adverse impact on the target variable since it yielded the highest net increase in the probability (35%) associated with patient safety errors (see Fig. 6a). In contrast, “*health and well‐being*” significantly reduced the probability of patient safety errors resulting in the highest net reduction in the probability (26%) associated with patient safety errors (see Fig. 6b). Other factors were found to be relatively noncritical. This implies that risk managers and policymakers may prioritize “health and well‐being” to realize significant benefits to the safety program. However, they may want to develop risk mitigation strategies to reduce the negative impact of “*bullying and harassment*” in the work environment.

We also conducted the Spearman's correlation test to establish the association between the two ranking schemes relative to the realization of the extreme states of individual factors (see Table VII). The two ranking schemes can help establish whether these organizational factors possess the same relative importance in relation to their vulnerability and resilience potential (Qazi & Khan, 2020). The association is found to be statistically nonsignificant, which implies that the optimal choice of risk mitigation strategies is contingent on individual factors’ vulnerability and resilience potential. In other words, an effective strategy can only be developed after exploring statistical dependencies among multidimensional factors and establishing the vulnerability and resilience potential of individual factors.

**Table VII:** Spearman's Correlation Analysis

Factor	Vulnerability‐Based Ranking Scheme	Resilience‐Based Ranking Scheme
Diversity	3	7
Immediate Managers	4.5	3
Quality of care	7	4.5
Safe environment—violence	8	6
Safety culture	6	8
Health and well‐being	2	1
Safe environment—b&h	1	2
Team working	4.5	4.5
Spearman's rank correlation coefficient	0.5843	
*p*‐value	0.1282	

## 5 DISCUSSION

Several organizational factors may have an impact on patient safety errors. Using the survey data, our results revealed that the organizational factors, including (X1) *equality, diversity, and inclusion*; (X2) *health and well‐being*; (X3) *immediate managers*; (X4) *quality of care*; (X5) *safety culture*; (X6) *team working*, (X7) *safe environment—bullying and harassment*; and (X8) *safe environment—violence*, have a statistically significant relationship with the patient safety errors. These factors were then fed into the BBN model to explore their interdependencies and relative importance towards patient safety errors. Based on the results, our model concludes that *health and well‐being* (X2), and *bullying and harassment in the work environment* (X7) are the two leading factors driving the number of reported errors and near misses affecting patient safety.

In the literature, these factors have been identified as contributing factors to the safety risks in various industries, including healthcare. Specifically, work‐related stress (e.g., burnout) is a trending topic that its role on patient and provider safety has been investigated widely in various healthcare settings (Tawfik et al., 2018; Yassi & Hancock, 2005). For instance, earlier studies showed that burnout contributes to emotional exhaustion and detached feelings towards patients (Koukoulaki et al., 2017; Lee et al., 2013). This may explain the strong relationship between *health and well‐being* factor and patient safety errors that we found in this study. Therefore, it can be recommended that increased psychological and emotional support to the provider may lead to improved patient safety.

Bullying and harassment in the work environment is an interesting factor to be found as a critical one affecting patient safety. Although it could be assumed that such an inappropriate environment would affect the healthcare providers’ psychological and physical health (Vessey, Demarco, & DiFazio, 2010) as well as financial costs (Kline & Lewis, 2019), it seems this also impacts patient safety outcomes (Chipps, Stelmaschuk, Albert, Bernhard, & Holloman, 2013; Felblinger, 2008; Johnson et al., 2019). For instance, a study identified *bullying* as an important factor and a “*hidden threat*” to patient safety and suggested strategies and initiatives to prevent such inappropriate behaviors (Longo & Hain, 2014). Further, recent empirical studies in Korean and Saudi hospitals also showed the relationship between bullying and nurse‐assessed patient safety outcomes (Al Omar, Salam, & Al‐Surimi, 2019; Oh, Uhm, & Yoon, 2016).

This study has made two unique contributions: (1) evaluating organizational factors and their influence on patient safety errors; and (2) adopting a data‐driven BBN approach to provide significant insights on the relative importance of the factors. To our knowledge, this is the first study evaluating a BBN‐based approach in this particular safety context. Identifying the importance of organizational factors is essential for organizations to drive safety improvement. In this study, it is important to note that the data‐driven BBN model has revealed some valuable insights regarding the factors’ relative importance, a central area of uncertainty in risk assessment (Aven, 2016). As earlier studies showed (McAfee & Brynjolfsson, 2012), there is an association between data use in the decision‐making process and operational performance. This study also highlighted the use of data by adopting the BBN model to support decision‐making in this interesting relationship between organizational factors and medical errors.

Further, it is noteworthy to mention that the relative importance of individual factors was established through change assessment in the probability distribution of patient safety errors regarding the realization of individual factors’ extreme states. In terms of the prediction capability, the chosen PC algorithm achieved a classification accuracy of 78% in a three‐state discretization scheme. Our findings revealed that healthcare organizations should improve organizational factors that their providers experience in the work environment to accelerate patient safety improvement. It is, nonetheless, important for not only organizations but also policymakers, such as governments, to support the important factors’ development with appropriate policy implementations (Jain, Leka, & Zwetsloot, 2018).

This study offers significant insights for risk practitioners and researchers to understand the impact of organizational factors on safety‐related performance measures. Starting with the most important organizational factors, healthcare organizations can improve their capabilities to reduce the number of errors affecting patient safety. Exploiting the data‐driven BBN model, individual organizations may also determine the most important factors affecting their safety outcomes. Exploring the interrelation between organizational factors and quantitative safety performances may offer further motivations to investigate such capabilities’ strengths and limitations. For instance, safety and risk managers may identify the underlying reasons behind work‐related stress and bullying to obtain positive results relative to patient safety. This study may also help to see the importance of data in this particular field. Although the survey is a commonly used approach for data collection and analysis in this specific context, they have not been used with the help of data‐driven approaches, such as BBN models, to demonstrate and analyze interrelations among the variables visually. The realistic results may encourage the healthcare managers to motivate data collection to improve the quality of analysis and knowledge gained.

## 6 CONCLUSIONS

This study identified organizational factors that have a significant effect on patient safety errors. Although a range of factors affect safety performance, it is vital for practicing risk and safety managers to prioritize them regarding their importance. This, therefore, requires the identification of the most important factors to add more value to the safety improvement process. Besides, the potential interactions among the organizational factors and safety errors are explored to provide interesting insights to both researchers and practitioners.

Utilizing the hospital‐level aggregate survey data from the U.K. hospitals, adoption of the BBN model revealed that *health and well‐being* (X2), and *bullying and harassment in the work environment* (X7) are the leading factors driving safety errors. Future studies may benefit from using the same methodology in a specific healthcare unit or organization. Future research may also evaluate and integrate other traditional or machine learning approaches to compare prediction capabilities and possible disparities in the relative importance of the organizational factors. It would also be interesting to collect data not only from providers but also from patients to investigate how patient experience is related to safety outcomes.

Our study has some limitations that may give a caution to interpret and generalize the results. First, this study is based on the hospital‐level aggregate data; therefore, specific units and hospitals may have differences in the relative importance ranking of the organizational factors. Second, the study is based on the data from the U.K. hospitals; therefore, the generalizability and transferability of the results to other industries and countries may be limited. Therefore, future research might also consider replicating this study in different healthcare settings or industries to help create a more holistic conclusion regarding the interdependencies between organizational factors and safety errors.

## Appendix

**Table AI:** Survey Themes (Organizational Factors and Patient Safety Errors) and Items (adapted from NHS SCC (2019))

ID	Survey Question
**X1**	**Equality, diversity, and inclusion**
q14	Does your organisation act fairly with regard to career progression/promotion, regardless of ethnic background, gender, religion, sexual orientation, disability, or age?
q15a	In the last 12 months, have you personally experienced discrimination at work from patients/service users, their relatives, or other members of the public?
q15b	In the last 12 months, have you personally experienced discrimination at work from the manager/team leader or other colleagues?
q28b	Has your employer made adequate adjustment(s) to enable you to carry out your work?
**X2**	**Health and well‐being**
q5h	The opportunities for flexible working patterns.
q11a	Does your organisation take positive action on health and well‐being?
q11b	In the last 12 months have you experienced musculoskeletal problems (MSK) as a result of work activities?
q11c	During the last 12 months, have you felt unwell as a result of work‐related stress?
q11d	In the last three months have you ever come to work despite not feeling well enough to perform your duties?
**X3**	**Immediate managers**
q5b	The support I get from my immediate manager.
q8c	My immediate manager gives me clear feedback on my work.
q8d	My immediate manager asks for my opinion before making decisions that affect my work.
q8f	My immediate manager takes a positive interest in my health and well‐being.
q8g	My immediate manager values my work.
q19g	My manager supported me to receive this training, learning or development.
**X4**	**Quality of care**
q7a	I am satisfied with the quality of care I give to patients/service users.
q7b	I feel that my role makes a difference to patients/service users.
q7c	I am able to deliver the care I aspire to.
**X5**	**Safety culture**
q17a	My organization treats staff who are involved in an error, near miss, or incident fairly.
q17c	When errors, near misses or incidents, are reported, my organization takes action to ensure that they do not happen again.
q17d	We are given feedback about changes made in response to reported errors, near misses and incidents.
q18b	I would feel secure raising concerns about unsafe clinical practice.
q18c	I am confident that my organization would address my concern.
q21b	My organization acts on concerns raised by patients/service users.
**X6**	**Team working**
q4h	The team I work in has a set of shared objectives.
q4i	The team I work in often meets to discuss the team's effectiveness.
**X7**	**Safe environment—bullying and harassment (b&h)**
q13a	In the last 12 months how many times have you personally experienced harassment, bullying or abuse at work from patients / service users, their relatives or other members of the public?
q13b	In the last 12 months how many times have you personally experienced harassment, bullying or abuse at work from managers?
q13c	In the last 12 months how many times have you personally experienced harassment, bullying or abuse at work from other colleagues?
**X8**	**Safe environment—violence**
q12a	In the last 12 months how many times have you personally experienced physical violence at work from patients / service users, their relatives or other members of the public?
q12b	In the last 12 months how many times have you personally experienced physical violence at work from managers?
q12c	In the last 12 months how many times have you personally experienced physical violence at work from other colleagues?
**X9**	**Target variable—patient safety errors**
q16b	In the last month, have you seen errors or near misses that could hurt patients?
